# Simplifying intensity‐modulated radiotherapy plans with fewer beam angles for the treatment of oropharyngeal carcinoma

**DOI:** 10.1120/jacmp.v8i2.2412

**Published:** 2007-04-11

**Authors:** Robert Takamiya, Brian Missett, Vivian Weinberg, Clayton Akazawa, Pam Akazawa, Andrea Zytkovicz, Mary Kara Bucci, Nancy Lee, Jeanne Quivey, Ping Xia

**Affiliations:** ^1^ Department of Radiation Oncology University of California–San Francisco San Francisco California U.S.A.; ^2^ Comprehensive Cancer Center University of California–San Francisco San Francisco California U.S.A.; ^3^ Biostatistics Core University of California–San Francisco San Francisco California U.S.A.

**Keywords:** oropharyngeal carcinoma, IMRT, beam angles

## Abstract

The first aim of the present study was to investigate the feasibility of using fewer beam angles to improve delivery efficiency for the treatment of oropharyngeal cancer (OPC) with inverse‐planned intensity‐modulated radiation therapy (IP‐IMRT). A secondary aim was to evaluate whether the simplified IP‐IMRT plans could reduce the indirect radiation dose. The treatment plans for 5 consecutive OPC patients previously treated with a forward‐planned IMRT (FP‐IMRT) technique were selected as benchmarks for this study. The initial treatment goal for these patients was to deliver 70 Gy to ≥95% of the planning gross tumor volume (PTV‐70) and 59.4 Gy to ≥95% of the planning clinical tumor volume (PTV‐59.4) simultaneously. Each case was re‐planned using IP‐IMRT with multiple beam‐angle arrangements, including four complex IP‐IMRT plans using 7 or more beam angles, and one simple IMRT plan using 5 beam angles. The complex IP‐IMRT plans and simple IP‐IMRT plans were compared to each other and to the FP‐IMRT plans by analyzing the dose coverage of the target volumes, the plan homogeneity, the dose–volume histograms of critical structures, and the treatment delivery parameters including delivery time and the total number of monitor units (MUs). When comparing the plans, we found no significant difference between the complex IP‐IMRT, simple IP‐IMRT, and FP‐IMRT plans for tumor target coverage (PTV‐70: p=0.56; PTV‐59.4: p=0.20). The plan homogeneity, measured by the mean percentage isodose, did not significantly differ between the IP‐IMRT and FP‐IMRT plans (p=0.08), although we observed a trend toward greater inhomogeneity of dose in the simple IP‐IMRT plans. All IP‐IMRT plans either met or exceeded the quality of the FP‐IMRT plans in terms of dose to adjacent critical structures, including the parotids, spinal cord, and brainstem. As compared with the complex IP‐IMRT plans, the simple IP‐IMRT plans significantly reduced the mean treatment time (maximum probability for four pairwise comparisons: p=0.0003). In conclusion, our study demonstrates that, as compared with complex IP‐IMRT, simple IP‐IMRT can significantly improve treatment delivery efficiency while maintaining similar target coverage and sparing of critical structures. However, the improved efficiency does not significantly reduce the total number of MUs nor the indirect radiation dose.

PACS number: 87.53.tf

## I. INTRODUCTION

Inverse‐planned intensity‐modulated radiation therapy (IP‐IMRT) in the treatment of head‐and‐neck carcinoma successfully improves target coverage while sparing the surrounding critical organs.^(^ ^1^ ^–^ ^4^ ^)^ Large‐scale clinical implementation of IP‐IMRT for patients with head‐and‐neck cancer might be hindered by the complexity of treatment planning and the prolonged treatment time, especially in centers using a static multileaf collimator (MLC). The increased treatment time might be considered attributable to “overly” modulated intensity beam profiles and the use of a large number of beams in IP‐IMRT plans. Forward‐planned IMRT plans (FP‐IMRT), with a shorter treatment time, are often used for very ill patients or for patients who can not tolerate prolonged treatment time. However, FP‐IMRT has the disadvantage of heavy dependence on the expertise of the planner to keep all sensitive structures below tolerance.

Complex IP‐IMRT plans using multiple beam angles and complex intensity modulation not only result in a prolonged treatment time, they also reduce “radiation efficiency” because of the use of many small segments (in static IMRT delivery) and numerous narrow openings (in dynamic IMRT delivery). Inefficient radiation subjects patients to unnecessary exposure because of the increased “indirect” radiation contribution from scatter and leakage dose. Mohan et al. showed that indirect radiation adversely affects the accuracy of treatment delivery.^(5)^ Biologically, the increased exposure from IP‐IMRT may increase the frequency of radiation‐induced secondary malignancies. Hall et al. recently showed that the transition from three‐dimensional (3D) conformal radiation therapy to IP‐IMRT results in a larger volume of normal tissue being exposed to a low dose of radiation, which is estimated to increase the incidence of secondary cancers from 1% to 1.75% at 10 years.^(6)^

Radiation efficiency and treatment‐plan quality are closely tied to beam angle arrangements and the number of beams used. When pressured to produce higher quality IP‐IMRT plans, a simple solution for the dosimetrist or physicist is to increase the number of beams. In addition, when an institution first implements IP‐IMRT, a tendency to use many equally spaced beam angles is seen. In many cases, too many gantry angles may be used. Research into computer optimization of beam‐angle selection has not proved clinically useful because of the large search space and its dependence on a specific clinical situation. Some researchers indicate that, as long as sufficient numbers of beam angles are used, the selection of beam angles in IMRT is not important.^(^ ^7^ ^,^ ^8^ ^)^

Besides beam angle selection, an optimal IP‐IMRT plan critically depends on the proper dose–volume constraints being assigned to the sensitive structures at risk. Improperly specified dose constraints may lead to an inferior plan despite computer optimization or properly selected gantry angles. Using the FP‐IMRT technique as a benchmark, the present study aimed to investigate the feasibility of using fewer beam angles in the treatment of oropharyngeal cancer with IP‐IMRT plans, achieving improved treatment delivery efficiency while adequately treating the tumor volume and sparing critical structures. In addition, the impact of simplified IP‐IMRT plans on indirect radiation was also evaluated.

## II. METHODS AND MATERIALS

### A. Patient selection and planning system

Five consecutive cases of OPC (T2N2A right tonsil, T2N2B left tonsil, T2N1 right tonsil, T2N2C base of tongue, and T2N2A base of tongue) originally treated between February and October 2002 with definitive radiation therapy planned using the FP‐IMRT technique developed at our institution^(9)^ were selected for this study. The FP‐IMRT plan for each patient served as a benchmark for evaluating all IP‐IMRT plans. All patients were planned with a commercial treatment planning system (Pinnacle 6.2: Philips Medical Systems, Andover, MA) capable of generating FP‐IMRT and IP‐IMRT plans, allowing us to directly compare plans using the same dose‐calculation algorithm.

### B. Planning goals and acceptance criteria

The treatment goal for each patient was to deliver a dose of 70 Gy in 33 fractions to at least 95% of the planning gross tumor volume (GTV)—designated the PTV‐70—while simultaneously treating 95% of the planning clinical tumor volume (CTV) with a dose of 59.4 Gy—designated the PTV‐59.4. The plan acceptance criteria were developed based on a previous review of a group of 23 IP‐IMRT clinical plans that had been generated for OPC patients with the Corvus planning system (NOMOS Division, North American Scientific, Chatsworth, CA).

Briefly, these plans typically used from 7 to 9 coplanar 6‐MV photon beams to treat the primary tumor and regional lymph nodes. The dose–volume histograms from these plans were analyzed for defined endpoints, depending on the characteristics of each sensitive structure. For parallel sensitive structures such as the parotids, the mean dose to the volume was used as the endpoint; for serial sensitive structures, the maximum dose to 1 cm^3^ of the structure's volume was used as the endpoint. Tables 1 and 2 list average endpoint data for the targets and sensitive structures from these IMRT plans—data that subsequently served as planning acceptance criteria for the present study.

### C. FP‐IMRT plans

The FP‐IMRT planning technique was developed and used in our clinic between 1995 and 2003. The details of this planning technique have been described elsewhere.^(9)^ Beam‐split FP‐IMRT plans were used to treat the primary and upper‐neck nodal regions. The lower‐neck and supraclavicular nodes were treated with a split‐beam anterior field matched to the inferior border of the FP‐IMRT plan at the isocenter.

Typically, 7 beam angles were used, including an anterior, 2 lateral, 2 anterior oblique, and 2 posterior oblique beams, with up to 3 beam shapes per angle, called “MLC segments.” The shape of each segment was manually designed, and the associated weights were adjusted through manual iterations. Depending on the particular case, the segments used in a given angle were tailored to maximize coverage of the target while minimizing normal‐tissue exposure. The first segment was constructed to treat both the PTV‐70 and the PTV‐59.4 with a margin of 5 mm to account for penumbra, blocking the spinal cord. The second segment was constructed to treat both the PTV‐70 and PTV‐59.4 at the same angle, without shielding the spinal cord. The third segment was constructed to treat only the PTV‐70 with a 5‐mm margin to account for penumbra. For this latter PTV‐70 boost segment, the spinal cord and brainstem were both shielded. Similar principles were applied to the other beam directions.

**Table 1:** Average endpoint doses (D) and endpoint volumes (V) for tumor targets

	Volume (cm^3^)	$D_{99}$ (Gy)	$D_{95}$ (Gy)	$D_{1\text{cm}3}$ (Gy)	$V_{93}$ (cm^3^)
PTV‐70	76.7±47.3	69.3±1.4	71.2±1.5	80.2±2.6	0.1±0.1
PTV‐59.4	690.4±274.1	54.3±4.7	60.6±2.7	80.8±2.0	9.0±4.8

PTV−70= planning gross target volume, 70‐Gy dose; PTV−59.4= planning clinical tumor volume, 59.4‐Gy dose; $V_{93}=$ volume receiving less than 93% of the prescribed dose; $D_{x\%}=$ dose to *x%* of the volume; $D_{1cm3}=$ maximum dose to 1 cm^3^ of the volume.

**Table 2:** Average endpoint doses (D) for sensitive structures

	$D_{1cm3}$ (Gy)	$D_{1\%}$ (Gy)	Mean *D* (Gy)	$D_{50\%}$ (Gy)
Spinal cord	42.6±3.5	40.2±3.8		
Brainstem	43.5±9.8	40.1±10.1		
Mandible	71.6±2.9	67.7±3.0		
Parotid			26.1±3.2	23.5±3.5

$D_{x\%}=$ maximum dose to *x%* of the volume; $D_{1cm3}=$ maximum dose to 1 cm^3^ of the volume.

### D. IP‐IMRT plans

For comparison purposes, all IP‐IMRT plans used split beams to treat the primary and upper‐neck nodal regions, as the FP‐IMRT plans did. Five different coplanar beam angle arrangements were examined, as listed in Table 3. The first beam‐angle configuration consisted of 9 equally spaced beams (the most prevalent beam arrangement found in the literature for head‐and‐neck IMRT). The second was an 8‐beam‐angle arrangement, based on our early experience of inverse planning. The third was a 7‐beam‐angle arrangement selected by an experienced dosimetrist and derived from FP‐IMRT. The fourth was a posterior‐weighted 7‐beam‐angle configuration based on a Memorial Sloan–Kettering technique described by Hunt et al.^(10)^ The fifth was a simple 5‐beam‐angle arrangement (including 1 anterior field, 2 anterior‐oblique fields, and 2 posterior‐oblique fields) to test whether a reduction in the number of beams was feasible. A template of planning dose constraints was constructed based on our previous planning experience in the Corvus planning system and was adapted for the Pinnacle planning system as shown in Table 4. For each patient, fine adjustment was required to achieve the treatment goals.

**Table 3:** The various beam angle arrangements

Beam	Angle (degrees)								
I	0	40	80	120	160	200	240	280	320
II	0	30	90	130	230	260	290	330	
III	0	60	90	150	210	270	300		
IV	90	120	150	180	210	240	270		
V	0	65	130	230	295				

**Table 4:** Planning dose constraint template

Region of interest	Type	Target (cGy)	% Volume	Weight
PTV‐70	Maximum dose	7500		20
PTV‐70	Minimum DVH	7000	98	25
PTV‐70	Minimum dose	6000		1
PTV‐59.4	Maximum dose	7000		12
PTV‐59.4	Minimum DVH	5940	98	25
PTV‐59.4	Minimum dose	5700		1
LT‐parotid	Maximum DVH	5400	15	1
LT‐parotid	Maximum DVH	2200	40	3
RT‐parotid	Maximum DVH	5400	15	1
RT‐Parotid	Maximum DVH	2200	40	3
Spinal cord	Maximum dose	4200		7
Brainstem	Maximum DVH	4100	1	3
Tongue	Maximum DVH	5500	10	1
Mandible	Maximum dose	6700		2

PTV−70= planning gross target volume, 70 Gy dose; PTV−59.4= planning clinical tumor volume, 59.4 Gy dose; DVH= dose–volume histogram; LT=Left; RT=right.

All IP‐IMRT plans were converted to MLC shapes using the IMFAST leaf‐sequencer provided by the Pinnacle treatment planning system. The conversion parameters used were 7 intensity levels, 3 extraction levels, and 1 MU minimal exposure per segment. Because plan quality is known to deteriorate after MLC conversion, each IP‐IMRT plan underwent an additional segment‐weight computer optimization.

### E. Plan comparison

All plans were compared based on defined multiple endpoint doses and volumes as shown in Table 1. Because mean doses were not provided by the planning system version used for the present study, the dose to 50% of the volume $\left(D_{50}\right)$ was used as an endpoint for the parotid glands, this dose being slightly different from the mean dose endpoint defined in Table 1. The parotid volume analysis is based on the entire volume, regardless of overlap with the tumor target.

The treatment efficiency was estimated by the delivery time, excluding patient setup time. Using Siemens linear accelerators (Primus: Siemens Medical Solutions, Concord, CA), IMRT plans can be delivered automatically from one beam angle to the next beam angle, including all segments within each beam, without therapist intervention. The delivery time depends on the total number of segments (the so‐called control points), total number of MUs, and the number of beam angles. Based on observation in our clinics, the beam pause time between segments is 7 seconds, and the beam pause time between beam angles is 14 seconds. The dose rate is 300 MU per minute for a 6‐MV photon beam. The delivery time in seconds, therefore, was estimated based on the formula

$$\text{Total}\;\text{Delivery}\;\text{Time}\;(\sec)=7(\sec)•(N_{\text{seg}}-1)+14(\sec)•(N_{gantry}-1)+60(\sec/\min)•\frac{MU_{total}}{300MU/\min}\;\;,$$

where $N_{seg}$ is the number of segments, $N_{\text{gantry}}$ is the number of gantry angles, and ${\text{MU}}_{\text{total}}$ is the total number of MUs in the plan.

### F. Statistical analysis

Descriptive statistics (mean, standard deviation, proportion) were calculated for each selected endpoint dose and for delivery parameters (total number of MUs) of each planning method. Analysis of variance for repeated measures was used to analyze the mean difference between the FP‐IMRT and the IP‐IMRT methods when the various beam‐angle arrangements were applied. Linear contrast statements were included for specific predefined comparisons (for example, simple IP‐IMRT vs. FP‐IMRT). Post hoc pairwise comparisons were performed using the Newman–Keuls test. Probability values less than 0.05 were considered to be statistically significant; however, the results are considered exploratory because of the small sample size.

## III. RESULTS

### A. Plan comparison

All IP‐IMRT plans with 5 different beam configurations accomplished excellent tumor coverage while restricting dose to the critical structures. In terms of tumor coverage, we observed no difference between the complex IP‐IMRT plans (from 7 to 9 beam angles), the simple IP‐IMRT plans (5 beam angles), and the FP‐IMRT plan (PTV‐70: p=0.56; PTV‐59.4: p=0.20). Considering all methods, the mean $V_{70}$ (percentage volume receiving more than 70 Gy) for the PTV‐70 ranged from 97.7% to 98.8%, and the mean $V_{59.4}$ for the PTV‐59.4 ranged from 94.6% to 96.1%, demonstrating excellent target coverage.

**Figure 1:**
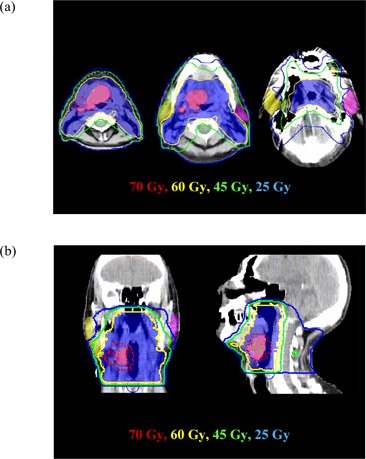
(a) Selected isodose lines (from 70 Gy to 25 Gy) displayed in various axial images from a simple inverse‐planned intensity‐modulated radiation therapy (IP‐IMRT) plan. (b) The same isodose lines displayed in coronal and sagittal images from the same simple IP‐IMRT plan. The pink color wash encompassing the blue color wash is the planning gross target volume, 70‐Gy dose (PTV‐70), and the blue color wash is the planning clinical tumor volume, 59.4‐Gy dose (PTV‐59.4).

Plan homogeneity was measured by the percentage ratio of the prescription dose that covered >95% of the PTV‐70 to the maximum point dose of the plan, called a “percentage isodose.” The mean percentage prescription isodose for all 5 patients did not significantly differ for the 6 treatment plans (five IP‐IMRT plans and one FP‐IMRT plan; p=0.08), with the mean percentage isodose being lowest for the simple IMRT plans (86.0%) as compared with the others (87.6%−88.6%). Fig. 1(a,b) shows isodose distributions for selected axial, sagittal, and coronal images of a simple IP‐IMRT plan.

### B. Sensitive structures

**Figure 2:**
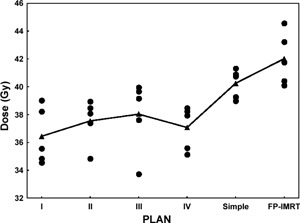
Distributions of the maximum dose to 1 cm^3^ of the spinal cord by four complex inverse planned, intensity‐modulated radiation therapy (IP‐IMRT) techniques (I–IV), the simple IP‐IMRT technique, and the forward‐planned IMRT (FP‐IMRT) technique for each patient (filled circles). The filled triangles represent the mean endpoint dose, averaged over the 5 patient plans.

For all patients, the mean maximum dose to 1 cm^3^ of the spinal cord ranged from 33.7 Gy to 40.0 Gy for all complex IP‐IMRT plans, from 39.44 Gy to 41.3 Gy for simple IP‐IMRT plans, and from 41.0 Gy to 44.6 Gy for FP‐IMRT plans. Although the mean maximum dose to the spinal cord showed a significant improvement with the complex IP‐IMRT techniques (beam configurations I–IV) over the simple IP‐IMRT (beam configuration V; post hoc pairwise comparisons, simple IMRT vs. complex IMRT: p=0.01, 0.03, 0.04, and 0.04 respectively), the mean maximum dose to the spinal cord in simple IP‐IMRT plans was still below the conventional tolerance of 45 Gy. We observed no significant difference in the mean maximum dose to the spinal cord between the simple IP‐IMRT plans and the FP‐IMRT plans (p=0.10). Fig. 2 shows the maximum dose to 1 cm^3^ of the spinal cord by technique.

**Figure 3:**
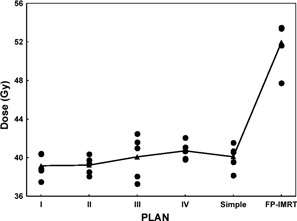
Distributions of the maximum dose to 1 cm^3^ of the brainstem by four complex inverse‐planned intensity‐modulated radiation therapy (IP‐IMRT) techniques (I–IV), the simple IP‐IMRT technique, and the forward‐planned IMRT (FP‐IMRT) technique for each patient (filled circles). The filled triangles represent the mean endpoint dose averaged over 5 patient plans.

For the brainstem, we observed no difference in the mean maximum dose to 1 cm^3^ of the volume $\left(D_{1\text{cm}3}\right)$ between the simple and each complex IP‐IMRT technique (post hoc pairwise comparisons, simple IMRT vs. complex IMRT: p=0.70, 0.48, 0.99, and 0.57 respectively), but we detected a significant improvement in mean $D_{1cm3}$ to the brainstem with the simple IP‐IMRT technique as compared with the FP‐IMRT technique (p=0.0001). Among the plans, the mean $D_{1cm3}$ of the brainstem ranged from 37.3 Gy to 42.5 Gy for complex IP‐IMRT plans, from 38.24 Gy to 41.5 Gy for simple IP‐IMRT plans, and from 47.75 Gy to 53.5 Gy for FP‐IMRT plans. Fig. 3 shows the distribution of dose to $D_{1cm3}$ of the brainstem by technique.

**Figure 4:**
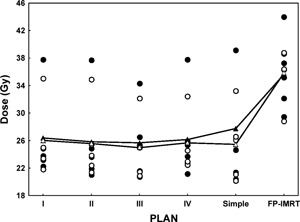
Distributions of the dose to 50% of the left and right side of the parotid glands by four inverse‐planned intensity‐modulated radiation therapy (IP‐IMRT) techniques (I–IV), the simple IP‐IMRT technique, and the forward‐planned IMRT (FP‐IMRT) technique for each patient (open and filled circles). The open and filled triangles represent the mean endpoint doses for both sides, averaged over the 5 patient plans.

In terms of parotid sparing, we again observed no difference between the complex and simple IP‐IMRT plans [post hoc pairwise comparisons, simple IMRT vs. complex IMRT (I–IV): p=0.77, 0.87, 0.88, and 0.91 respectively], but we detected a significant advantage for the simple IP‐IMRT technique over the FP‐IMRT technique, resulting in a lower mean $D_{50}(p=0.0002)$. Fig. 4 plots the dose to 50% of each parotid gland. Except for 1 patient, who had locally advanced disease with invasion into the parotid glands, the dose to 50% of each parotid gland was less than 30 Gy in all of the IP‐IMRT plans. For all patient plans, the mean dose to 50% of the parotid glands (left/right) ranged from 25.68/24.97 Gy to 27.75/26.02 Gy for IP‐IMRT, while FP‐IMRT delivered mean doses to the parotids of 35.61/35.81 Gy. It should be noted that the mean $D_{50}$ for these patients was slightly higher than averaged $D_{50}$ provided in Table 1, but we still considered that these IMRT plans met acceptance criteria, because the two planning systems have an inherent discrepancy with regard to the reporting of parotid doses. With the Corvus planning system, the parotid doses reported in Table 1 were the doses to truncated parotid volumes, because the system does not allow any two volumes to overlap. The Pinnacle planning system allows overlapping volumes, thus the reported $D_{50}$ in Fig. 4 reflects dose to the entire parotid volume, not the truncated parotid volume.

### C. Treatment delivery

The simple IP‐IMRT method required a significantly shorter treatment delivery time than did each of the four complex plans (p=0.0001, 0.0002, 0.0003, and 0.0002 respectively). The mean delivery time for simple IP‐IMRT was 13.7 minutes; the complex IP‐IMRT methods I–IV required 21.9, 20.7, 17.6, and 17.4 minutes respectively. Pairwise comparisons showed that complex IP‐IMRT techniques with more gantry angles (IP‐IMRT I and II) required significantly more delivery time than did the techniques with fewer gantry angles (IP‐IMRT III and IV), a linear contrast of p=0.002.

With the step‐and‐shoot IMRT delivery method using static MLC, the total number of segments in a plan is linearly correlated with the delivery time. The mean total number of segments is significantly different between the IP‐IMRT configurations (p<0.0001), with simple IP‐IMRT having fewer segments on average than do each of the complex IP‐IMRT plans. The mean number of segments for simple IP‐IMRT was 66.8; complex IP‐IMRT methods I–IV required 124.6, 90.6, 91.2, and 109.8 segments respectively.

**Figure 5:**
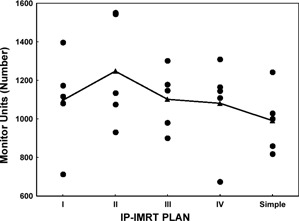
The distribution of total monitor units (MUs) for each patient plan by four complex inverse‐planned intensity‐modulated radiation therapy (IP‐IMRT) techniques (I–IV) and the simple IP‐IMRT technique (filled circles). The filled triangles represent the mean total number of MUs, averaged over the 5 patient plans.

The total MUs used in a plan are important because they contribute to the leakage and scatter dose to the patient's body. Although the average MUs used in simple IP‐IMRT plans were not significantly different from those used in the complex IP‐IMRT plans (linear contrast for IP‐IMRT: p=0.06), simple IP‐IMRT plans tended to use fewer MUs. Fig. 5 shows the distribution of total MUs in each patient plan for the four complex IP‐IMRT techniques and the simple IP‐IMRT technique. As expected, FP‐IMRT required the lowest number of segments and resulted in a significantly lower average total MUs (p<0.0002 for each pairwise comparison).

## IV. DISCUSSION

The significantly increased treatment times with complex IMRT plans have been a concern of physicians, therapists, and patients. The prolonged treatment times and the restrictive immobilization devices are particularly challenging for very sick patients. The dosimetric consequences of patient movement during treatment may trade off the dosimetric advantages of complex IP‐IMRT plans. Furthermore, complex IMRT plans that contain many segments with very small MUs and irregular shapes may also cause relatively large uncertainties in delivered doses. Biologically, the efficacy of radiation therapy may be reduced with prolonged treatment delivery. Thus, given all these considerations, we strive to implement IP‐IMRT in the most efficient fashion.

A common and devastating sequela of radiotherapy for most head‐and‐neck patients is xerostomia, which is directly correlated with the mean dose received by the parotid glands.^(^ ^11^ ^–^ ^16^ ^)^ Our study showed that the main benefit of IP‐IMRT over FP‐IMRT is the ability to spare the parotid glands—at the price of an increase in the total MUs as compared with the MUs in FP‐IMRT. With IP‐IMRT, the gain in sparing of the parotid glands outweighed the increased MUs. On the other hand, because complex IP‐IMRT plans did not further improve parotid sparing over a simple IP‐IMRT plan, the latter plan was desirable: it required less treatment time, a fewer number of segments, and somewhat fewer MUs.

We also found that simply reducing the number of beam angles does not significantly reduce indirect radiation (including scatter and leakage dose), as measured by the total number of MUs per treatment. To further reduce indirect radiation, drastic changes to inverse planning should be sought—for example, use of the direct‐aperture optimization method (DAO) as proposed by Shepard et al.,^(17)^ who, based on a variety of test cases, found a significant reduction in both the number of segments and the number of MUs. Further clinical evaluation of this optimization method is necessary, and we are currently carrying out studies at our institution.

The present study has some limitations. One limitation is its lack of an exhaustive search for all possible 5‐beam‐angle arrangements for the patients. A new study with that goal is currently in progress. As mentioned earlier, other methods of simplifying IMRT plans are available—for example, restricting the total number of segments for each plan. While the present study was being conducted, the option of presetting a total number of segments was not available. A preset option is now available in some planning systems, but it is not universally available to all users. Thus, the present study still has value.

## V. CONCLUSIONS

Delivery efficiency can be improved over complex IP‐IMRT plans by using simple IP‐IMRT plans with 5 beam angles, while maintaining similar tumor coverage and dose sparing to the adjacent critical structures. However, the improved efficiency does not significantly reduce the number of MUs, and thus the scatter and leakage dose is not significantly reduced. To further reduce indirect radiation dose, new optimization methods such as direct‐aperture optimization may provide a solution. Clinical validation of these optimization methods is necessary.

## References

[1] Xia P , Fu KK , Wong GW , Akazawa C , Verhey LJ . Comparison of treatment plans involving intensity‐modulated radiotherapy for nasopharyngeal carcinoma. Int J Radiat Oncol Biol Phys. 2000; 48 (2): 329–337. 10974445 10.1016/s0360-3016(00)00585-x

[2] Cozzi L , Fogliata A , Bolsi A , Nicolini G , Bernier J . Three‐dimensional conformal vs. intensity‐modulated radiotherapy in head‐and‐neck cancer patients: comparative analysis of dosimetric and technical parameters. Int J Radiat Oncol Biol Phys. 2004; 58 (2): 617–624. 14751535 10.1016/j.ijrobp.2003.09.059

[3] Pirzkall A , Carol M , Lohr F , Hoss A , Wannenmacher M , Debus J . Comparison of intensity‐modulated radiotherapy with conventional conformal radiotherapy for complex‐shaped tumors. Int J Radiat Oncol Biol Phys. 2000; 48 (5): 1371–1380. 11121636 10.1016/s0360-3016(00)00772-0

[4] Chao KS , Ozyigit G , Blanco AI , et al. Intensity‐modulated radiation therapy for oropharyngeal carcinoma: impact of tumor volume. Int J Radiat Oncol Biol Phys. 2004; 59 (1): 43–50. 15093897 10.1016/j.ijrobp.2003.08.004

[5] Mohan R , Arnfield M , Tong S , Wu Q , Siebers J . The impact of fluctuations in intensity patterns on the number of monitor units and the quality and accuracy of intensity modulated radiotherapy. Med Phys. 2000; 27 (6): 1226–1237. 10902551 10.1118/1.599000

[6] Hall EJ , Wuu CS . Radiation‐induced second cancers: the impact of 3D‐CRT and IMRT. Int J Radiat Oncol Biol Phys. 2003; 56 (1): 83–88. 12694826 10.1016/s0360-3016(03)00073-7

[7] Pugachev A , Xing L . Incorporating prior knowledge into beam orientation optimization in IMRT. Int J Radiat Oncol Biol Phys. 2002; 54 (5): 1565–1574. 12459386 10.1016/s0360-3016(02)03917-2

[8] Stein J , Mohan R , Wang X , et al. Number and orientations of beams in intensity‐modulated radiation treatments. Med Phys. 1997; 24 (2): 149–160. 9048355 10.1118/1.597923

[9] Lee N , Akazawa C , Akazawa P , et al. A forward‐planned treatment technique using multisegments in the treatment of head‐and‐neck cancer. Int J Radiat Oncol Biol Phys. 2004; 59 (2): 584–594. 15145180 10.1016/j.ijrobp.2004.02.005

[10] Hunt MA , Zelefsky MJ , Wolden S , et al. Treatment planning and delivery of intensity‐modulated radiation therapy for primary nasopharynx cancer. Int J Radiat Oncol Biol Phys. 2001; 49 (3): 623–632. 11172942 10.1016/s0360-3016(00)01389-4

[11] Radiation Therapy Oncology Group . RTOG 0022: phase I/II study of conformal and intensity modulated irradiation for oropharyngeal cancer. Eisbruch A , Chao KS , Garden AS , co‐chairs. Philadelphia (PA): Radiation Therapy Oncology Group; 2001.

[12] Eisbruch A , Ten Haken RK , Kim HM , Marsh LH , Ship JA . Dose, volume, and function relationships in parotid salivary glands following conformal and intensity‐modulated irradiation of head and neck cancer. Int J Radiat Oncol Biol Phys. 1999; 45 (3): 577–587. 10524409 10.1016/s0360-3016(99)00247-3

[13] Chao KS , Deasy JO , Markman J , et al. A prospective study of salivary function sparing in patients with head‐and‐neck cancers receiving intensity‐modulated or three‐dimensional radiation therapy: initial results. Int J Radiat Oncol Biol Phys. 2001; 49 (4): 907–916. 11240231 10.1016/s0360-3016(00)01441-3

[14] Eisbruch A , Kim HM , Terrell JE , Marsh LH , Dawson LA , Ship JA . Xerostomia and its predictors following parotid‐sparing irradiation of head‐and‐neck cancer. Int J Radiat Oncol Biol Phys. 2001; 50 (3): 695–704. 11395238 10.1016/s0360-3016(01)01512-7

[15] Chao KS . Protection of salivary function by intensity‐modulated radiation therapy in patients with head and neck cancer. Semin Radiat Oncol. 2002; 12 (1 suppl 1): 20–25. 11917280 10.1053/srao.2002.31359

[16] Lin A , Kim HM , Terrell JE , Dawson LA , Ship JA , Eisbruch A . Quality of life after parotid‐sparing IMRT for head‐and‐neck cancer: a prospective longitudinal study. Int J Radiat Oncol Biol Phys. 2003; 57 (1): 61–70. 12909216 10.1016/s0360-3016(03)00361-4

[17] Shepard DM , Earl MA , Li XA , Naqvi S , Yu C . Direct aperture optimization: a turnkey solution for step‐and‐shoot IMRT. Med Phys. 2002; 29 (6): 1007–1018. 12094970 10.1118/1.1477415

